# Characterization of a Cytomegalovirus-Specific T Lymphocyte Product Obtained Through a Rapid and Scalable Production Process for Use in Adoptive Immunotherapy

**DOI:** 10.3389/fimmu.2020.00271

**Published:** 2020-02-25

**Authors:** Marta Grau-Vorster, María López-Montañés, Ester Cantó, Joaquim Vives, Irene Oliver-Vila, Pere Barba, Sergi Querol, Francesc Rudilla

**Affiliations:** ^1^Cell Therapy Service, Banc de Sang i Teixits, Barcelona, Spain; ^2^Transfusion Medicine Group, Vall d'Hebron Research Institute, Universitat Autònoma de Barcelona, Barcelona, Spain; ^3^Musculoskeletal Tissue Engineering Group, Vall d'Hebron Research Institute (VHIR), Universitat Autònoma de Barcelona, Barcelona, Spain; ^4^Medicine Department, Universitat Autònoma de Barcelona, Barcelona, Spain; ^5^Hematology Department, Hospital Universitari Vall d'Hebron, Universitat Autònoma de Barcelona, Barcelona, Spain

**Keywords:** virus specific T lymphocytes (VST), antigen presenting cells (APC), adoptive immunotherapy, specificity, cytotoxicity, peripheral blood mononuclear cells (PBMC), alloreactivity

## Abstract

Immunosuppressed patients are susceptible to virus reactivation or *de novo* infection. Adoptive immunotherapy, based on virus-specific T lymphocytes (VST), can prevent or treat viral diseases. However, donor availability, HLA-compatibility restrictions, high costs, and time required for the production of personalized medicines constitute considerable limitations to this treatment. *Ex vivo* rapid and large-scale expansion of VST, compliant with current good manufacturing practice (cGMP) standards, with an associated cell donor registry would overcome these limitations. This study aimed to characterize a VST product obtained through an expansion protocol transferable to cGMP standards. Antigenic stimulus consisted of cytomegalovirus (CMV) pp65 peptide pool-pulsed autologous dendritic cells (DCs) derived from monocytes. G-Rex technology, cytokines IL-2, IL-7, and IL-15, and anti-CD3 and anti-CD28 antibodies were used for culture. At day 14 of cell culture, the final product was characterized regarding T cell subsets, specificity, and functionality. The final product, comprised mainly CD4^+^ and CD8^+^ T lymphocytes (49.2 ± 24.7 and 42.3 ± 25.2, respectively). The culture conditions made it possible to achieve at least a 98.89-fold increase in pp65-specific CD3^+^ IFN-γ^+^ cells. These cells were specific, as pp65-specific cytotoxicity was demonstrated. Additionally, in complete HLA mismatch and without the presence of pp65, alloreactivity resulted in <5% cell lysis. In conclusion, a cGMP scalable process for the generation of a large number of doses of CMV-specific cytotoxic T cells was successfully performed.

## Introduction

Immunodeficient patients are susceptible to infection by cytomegalovirus (CMV) and other viruses. Herpesviruses, such as CMV, are often asymptomatic or mild in healthy individuals. Nevertheless, in the context of immunocompromised patients, viral infections can be a severe cause of morbidity and mortality (1, 2). CMV infection rates indicate that the virus is common in most of the population and that incidence increases with age (3). The impaired immune systems of immunocompromised patients are unable to eradicate or limit the virus. Antiviral pharmacologic agents are effective against only some of these viruses; their use is costly, associated with significant toxicities and does not provide long-term protection (4, 5). Adoptive immunotherapy based on virus-specific T lymphocytes (VST) is therefore an attractive option, as T lymphocytes can confer long-term protection against the development of viral disease (6–8).

Donor lymphocyte infusion (DLI) is a conventional therapy for leukemia relapse after Hematopoietic Stem Cell Transplantation (HSCT) and it has also shown activity against virus infections in this setting (9, 10). But the use of non-specific lymphocytes to treat viral infection has the risk to develop graft-versus-host disease (GvHD) (11). In addition, DLI may not be effective when stem cell donors are seronegative as it is the case in umbilical cord blood transplantation. However, therapeutic alternative based on antigen-specific T cells is safe and effective, as it does not increase the risk of GvHD and shows overall response rates of 90% (12). Direct selection with peptide-multimers (13) or the cytokine capture system (14, 15) are currently used to obtain VST. Both procedures present certain limitations, such as the challenge of finding an available compatible and seropositive donor, and the amount of final product obtained, which defines the number of doses. While multimer selection presents the disadvantage of being limited to certain HLA types, the cytokine capture system will only select T cells producing a specific cytokine. Another hurdle of these methodologies is the very low frequency of CMV-specific T cells present in peripheral blood. According to Gamadia et al. (16), the frequency of CD8^+^ interferon gamma (IFN-γ) producing cells ranged between 0.18 and 0.80%, with similar observed CD4^+^ IFN-γ^+^ frequencies.

In order to create a VST bank, the cell expansion protocol must be well-established and must minimize the presence of alloreactive T cells in the final product, and the expanded T cell populations must contain both CD8^+^ and CD4^+^ cells to ensure an effective response to the infection (17, 18).

We describe a process for generating a single preparation of antiviral T lymphocytes (CD4^+^ and CD8^+^) that is consistently specific for immunodominant and subdominant antigens derived from CMV, a frequent cause of post-transplant morbidity or death. This approach uses a standardized mix of peptides focusing on pp65 protein, the major immunodominant CMV antigen, presented by mature monocyte-derived dendritic cells (moDC), and a combination of cytokines to promote the activation, survival, and expansion of T cells. After a bibliographic search of the media supplements most commonly used for lymphocyte expansion, cytokines IL-2, IL-7, and IL-15 (19–22) were selected for VST activation and expansion. Protocol optimization focused on reducing production time and maximizing product functionality. We also aimed to generate large amounts of cells to ensure the availability of several doses for a single patient if needed. Importantly, this method is readily adaptable to clinical implementation in compliance with current good manufacturing practice (cGMP) legislation and the product may be used as a safe and effective antiviral agent for patients at high risk of disease due to CMV infection.

## Materials and Methods

### PBMC Handling

Peripheral blood mononuclear cells (PBMC) were collected from the peripheral blood of 9 CMV^+^ seropositive healthy donors with donor informed consent and the corresponding approval of the ethics committee (complies with good clinical practice CPMP/ICH/135/95 and Real Decreto 1090/2015). PBMC isolation, cryopreservation, and thawing were performed as described in previous studies (23). Positive serology for CMV IgG was confirmed using chemiluminescence (Abbot, Abbot Park, Illinois, USA). PBMC from each donor were used to obtain CMV-specific VST, moDCs and phytohemagglutinin (PHA) lymphoblasts. The determination of cell concentration was performed by flow cytometry (Perfect-Count Microspheres™; Cytognos, Salamanca, Spain) or Neubauer chamber counting. The percentage of cell viability was determined either by 7-aminoactinomycin D (7AAD; BD Biosciences, San Jose, California, USA) staining or Trypan Blue (GE Healthcare, USA). Data was analyzed using CellQuest Pro software version 5.2.1 (BD Biosciences).

### Obtaining and Pulsing Dendritic Cells

Freshly isolated PBMC were initially seeded for 2 h to allow the adherence of monocytes. Non-adherent PBMC were then collected and cryopreserved. Monocytes were cultured for 8 days to obtain mature dendritic cells (DC), as described elsewhere (24). Mature DC were pulsed for 1–2 h at 37°C, 5% CO_2_, with 10 μg/mL of PepTivator® CMV pp65 (Miltenyi Biotec, Bergisch Gladbach, Germany), referred to here as pp65. PepTivator CMV pp65 is based on peptide pools of mainly 15-mer peptides with 11-amino acid (aa) overlap, covering the complete sequence of the pp65 protein of human cytomegalovirus. Pulsed-DC were used for PBMC coculture.

### Expansion Protocol

**Figure 1:**
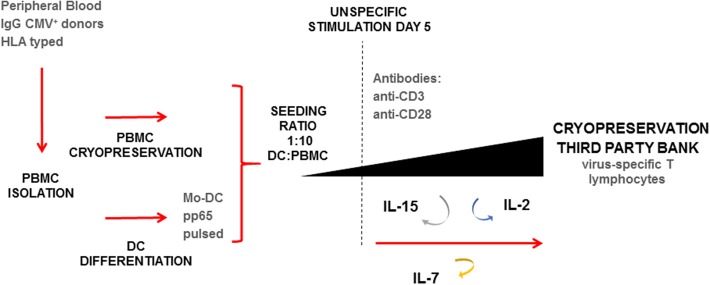
Expansion protocol schematic for the rapid generation of virus-specific T cells (VST). Starting material based on HLA typed peripheral blood IgG^+^ for CMV. Isolation of PBMC and selection of monocytes by plastic adherence. Non-adherent PBMC cryopreserved, and attached monocytes differentiated to DC. Seeding performed in a G-Rex device in a ratio 1:10 pp65-pulsed DC:thawed PBMC. Expansion lasted 14-day, with addition at day 5 of anti-CD3^+^ anti-CD28 antibodies, and IL-2, IL-7, IL-15. Renewal of cytokines was performed at days 5, 7, 9, and 12. At day 14 the final product was characterized and cryopreserved.

As shown in Figure 1, thawed PBMC were seeded at 0.25-1.0E+06 cells/cm^2^ at a DC:PBMC ratio of 1:10 in a G-Rex culture system (Wilson Wolf Manufacturing, New Brighton, MN). Fresh medium was composed of Roswell Park Memorial Institute GlutaMAX media (RPMI, Gibco Laboratories, NY, USA) supplemented with 10% (v/v) human AB serum (hSerAB) (Banc de Sang i Teixits, Barcelona, Spain). After 5 days of culture, the medium was completely replaced with RPMI+10% hSerAB supplemented with IL-2 120 U/mL (Miltenyi Biotec), IL-7 4,400 U/mL (R&D Systems, MN, USA), and IL-15 80 U/mL (Miltenyi Biotec). Moreover, monoclonal antibodies anti-CD3 (1 μg/mL) and anti-CD28 (2 μg/mL) were added (Biolegend, San Diego, California, USA). On days 7, 9, and 12, an aliquot of 100–300 μL was taken from each culture for cell counting and viability analysis, and fresh medium consisting of RPMI+10% hSerAB with the addition of IL-2 120 U/mL, IL-7 4,400 U/mL and IL-15 80 U/mL was added. Culture split was performed when cell density reached 8E+06 cells/cm^2^.

### Obtaining and Pulsing Lymphoblasts

Fresh or cryopreserved PBMC were adjusted to 2E+06 cells/mL and stimulated with PHA 5 μg/mL. After 24 h, cells were washed twice with Dulbecco's phosphate-buffered saline solution (PBS, Gibco Laboratories) by centrifugation at 340 g, RT, for 10 min. The solution was then adjusted again to 2E+06 cells/mL with RPMI+10%hSerAB and supplemented with IL2 (100 U/mL). At least 4 mL of this sample was seeded (2 mL/well) in a 24-well plate and the medium was replenished every 2–4 days. The culture was stored in the incubator at 37°C, 5% CO_2_ as long as G-Rex culture lasted. On the last day of culture, half of the blasts were pulsed with 1 μg/mL of pp65 peptide pool (Miltenyi) for 1–2 h at 37°C 5% CO_2_ and the other half were left non-pulsed. When blasts were used in an allogeneic context, samples were irradiated at 30 Gy for 15 min.

### PBMC Immunophenotype

For the phenotype analysis, PBMC were collected and stimulated with 1 μg/mL of pp65 for 6 h at 37°C, 5% CO_2_, at 1E+07 cells/mL. After 3 h of stimulation, 1 μg/mL of brefeldin A was added. The cells were then washed and permeabilized using Cytofix/Cytoperm solution (BD). Staining consisted of Live/Dead and monoclonal antibodies: CD45, CD8, CD3, IFN-γ, CD19, CD20, CD56, CD4, FOXP3, CD25, C-C chemokine receptor 7 type (CCR7), and CD45RA (Miltenyi Biotech).

For the analysis of the frequency of HLA-A^*^0201/NLVPMVATV CD8^+^ T cells, APC labeled Pro5 MHC Pentamer (Proimmune, Oxford, UK) was used. Staining was performed according to manufacturers' instructions.

Data acquisition was performed using a Miltenyi MACS Quant flow cytometer. Phenotype was analyzed using FlowJo software v10.

### Kinetics of Cytokine Secretion

Cryopreserved expanded cells were stimulated with CMV pp65 peptide pool (Miltenyi) 50 ng/mL at a cell concentration of 1E+06 cells/mL in a 96-well plate. Supernatant samples were collected at 0, 6, 10, 24, and 48 h. All samples were centrifuged at 1,000 g for 15 min at 4°C. To remove aggregates or debris, samples were centrifuged again at 10,000 g for 10 min at 4°C and stored at −80°C until analysis. Samples were thawed just once and kept on ice before assay. The Bio-Plex Pro™ Human Cytokine Th1/Th2 (Bio-Rad Laboratories, Hercules, CA, USA) was used for the determination of levels of 9 cytokines: IL-2, IL-4, IL-5, IL-10, IL-12 (p70), IL-13, IFN-γ, GM-CSF, and TNF-α in a multiplex assay using a Luminex 100IS analyzer (Luminex Corp. Austin. TX, USA) according to the manufacturer's instructions. Duplicates were tested for each sample. Data analysis was performed using Bioplex Manager Software v6.1 (Bio-Rad Laboratories Inc.).

### Enzyme-Linked Immunospot (ELISPOT)

ELISPOT (MABTECH, Nacka, Sweden) assay was performed in order to determine the specificity of T cells against CMV pp65 peptide by means of interferon gamma secretion. Cells were stimulated with 50 ng/mL pp65 peptide pool (Miltenyi) and left overnight at 2E+05 cells/well. Plates were read in an AID ELISPOT reader (AID GMBH, Strassberg, Germany) following manufacturer's instructions.

### CD107a Degranulation Assay

The final product was incubated for 4 h at 37°C with 10% CO_2_ in the presence of monensin (5 ng/mL, Sigma-Aldrich, Saint Louis, Missouri, USA) and anti-CD107a FITC (BD Biosciences Pharmingen) with PepTivator® CMV pp65 (Miltenyi). Stimulation with PHA (Sigma-Aldrich) was used as a positive control for degranulation. Unstimulated cells were used as a negative control for degranulation. Data acquisition was performed using a MACSQuant instrument (Miltenyi Biotech). Data analysis was performed using FlowJo software v10.

### Cytotoxicity Assay Based on Flow Cytometry

A cytotoxicity assay was performed to detect the manufactured lymphocytes' ability to attack either autologous or allogeneic lymphoblasts with or without presentation of CMV pp65 peptides (Miltenyi). Lymphoblasts were labeled with CFSE (CellTrace™ CFSE Cell Proliferation Kit; Invitrogen, Waltham, MA, USA), as already reported (23). Allogeneic and autologous non-pulsed lymphoblasts were stained with a high concentration of CFSE (2.5 μM), while pp65 peptide pool-pulsed lymphoblasts were labeled with a low concentration of CFSE (0.25 μM) (25). Fluorescence loss in the target population was monitored by means of flow cytometry in order to quantify the percentage of specific cytotoxicity (26). Two different ratios of target (T) and effector (E) cells were tested: T:E 1:5 and 1:10, maintaining 1E+04 target cells in both conditions. Triplicates were performed for each condition. Samples were seeded in a 96-well plate and were incubated for 4 and 24 h of coculture. Data acquisition was performed using a FACS Calibur instrument (BD). Data analysis was performed using FlowJo software v10.

Percentage of specific cytotoxicity was calculated using the ratio of pulsed and non-pulsed target cells without the presence of effector cells as a baseline, according to the following equation (27):

(1) $$\begin{array}{l} \text{Cell lysis }(\%)=100-\left[\text{100}*\frac{\text{Sample}(\text{CFSElow/CFSEhigh})}{\text{Baseline}(\text{CFSElow/CFSEhigh})}\right] \end{array}$$

### HLA Typing

HLA typing for all samples was performed using the next generation sequencing method. Briefly, DNA samples were amplified by means of multiplex PCR using an in-house strategy. After DNA library preparation (GenDX, Utrecht, The Netherlands), pooled samples were paired-end sequenced using a Miseq (Illumina, San Diego, California, USA) following manufacturer's instructions. Data analysis was performed using NGSengine software (GenDX) and IMGT HLA data versions 3.29.0–3.35.0.

### Data Analysis

Microsoft Office Excel® and GraphPad Prism 6 were used for the analysis of the results and for plot generation. Statistical significance was set at: ^*^p < 0.05, ^**^p < 0.01, and ^***^p < 0.001, Mann-Whitney test.

## Results

### Large Expansion of VST in 14-Day Coculture

**Figure 2:**
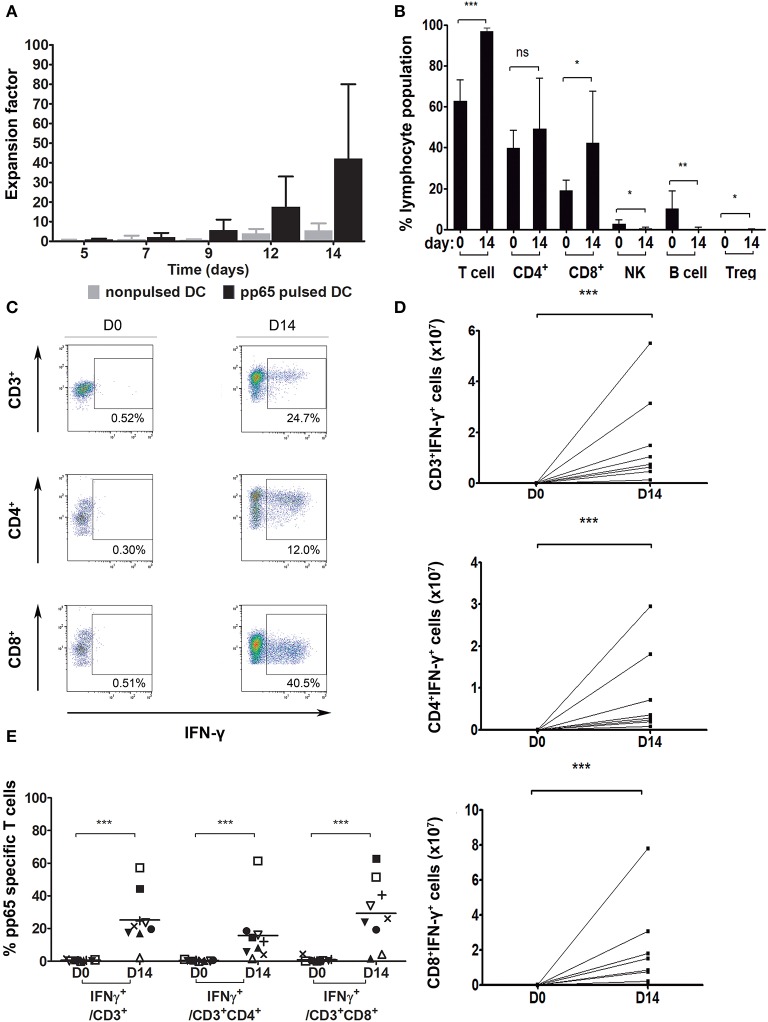
Pp65-specific T cell expansion. **(A)** Number of total cells at different time points during the 14-day culture. PBMC cocultured with: pp65 peptide pool-pulsed DC (black, *n* = 9) and nonpulsed DC (gray, *n* = 4). **(B)** Percentage of general cell subpopulations pre- (day 0) and post-expansion (day 14). T cells: CD3^+^, CD4^+^: CD3^+^CD4^+^, CD8^+^: CD3^+^CD8^+^, NK: CD3^−^CD56^+^, B cell: CD3^−^CD19^+^CD20^+^, Treg: CD3^+^CD4^+^CD25^+^FOXP3^+^ (*n* = 9). **(C)** Gating strategy for IFN-γ secreting cells from one representative donor. Dot plots show the response of T lymphocytes against pp65 antigen stimulation. **(D)** Number of VST at day 0 and after the 14-day culture (*n* = 8). **(E)** Percentage of IFN-γ secreting population cells at days 0 and 14 of culture (*n* = 9). *p < 0.05, **p < 0.01 and ***p < 0.001 (Mann-Whitney test).

Nine VST batches with pp65 specificity were manufactured from nine healthy CMV^+^ donors. We obtained a median of 43.3E+06 cells (min., 4.5E+06 cells; max., 100.1E+06 cells) from an initial seed of 1E+06 PBMC, representing an average 42.2-fold total expansion within 14 days. The growth kinetics of these cells is shown in Figure 2A. Cells grow slowly up to day 7 and start proliferating exponentially at day 9, with the highest proliferation at days 12–14 of culture (Figure 2A). More specifically, the overall expansion factor of CD3^+^ cells for PBMC cocultured with pulsed DC was an average of 49.3 ± 48.6 (median, 13.6; min, 6.7; max, 127.2; *n* = 9) while for PBMCs cocultured with non-pulsed DC, it was an average of 9.6 ± 6.3 (median, 8.6; min, 4.1; max, 17; *n* = 4).

### Final Product Mainly Comprising T Cells

The presence of a variety of cell populations in the final product (CD4^+^ T cells, CD8^+^ T cells, NK cells, B cells and Treg cells) was evaluated. Expanded cells largely consisted of CD3^+^ T cells (mean, 96.9 ± 1.9%) containing both CD4^+^ (mean, 49.2 ± 24.7%) and CD8^+^ (mean, 42.3 ± 25.2%) populations (Figure 2B). Compared to the phenotype of the initial product, significant differences were found in the CD3^+^ cell subset, which had largely expanded (*p* = 0.0004), with no changes among the CD4^+^ cell subset (*p* = 0.2891) but with a significant increase in the CD8^+^ cell population (*p* = 0.0315). B cells, NK cells, and Treg cells did not expand and, consequently, their presence decreased to almost undetectable levels in the final product (B cells, 0.3 ± 0.6%; *p* = 0.0013; NK cells, 0.5 ± 0.6%; *p* = 0.0225; and Treg cells, 0.1 ± 0.1%; *p* = 0.0395).

### Expanded VST Cells Show Antiviral Specificity Using IFN-γ

In order to test the lymphocytes' CMV specificity, cells were re-exposed to pp65-pepmix and activation was measured using IFN-γ intracellular production by flow cytometry. IFN-γ secretion of CD3^+^, CD3^+^CD4^+^, and CD3^+^CD8^+^ populations is shown in a representative dot plot (Figure 2C). In the independent expansions performed, at day 14 of coculture with pp65-pulsed moDC, the total number of CD3^+^IFN-γ^+^, CD3^+^CD4^+^IFN-γ^+^, and CD3^+^CD8^+^IFN-γ^+^ cells specific for CMV-pp65 was significantly higher compared to day 0 (Figure 2D) (*p* = 0.0002). Absolute values of CD3^+^IFN-γ^+^, CD4^+^IFN-γ^+^ and CD8^+^IFN-γ^+^ cells were calculated from day 14 with respect to day 0. The fold expansion results for CD3^+^IFN-γ^+^ cells were min, 98.89; max, 12,528; and median, 1480.18. The results for CD4^+^IFN-γ^+^ cells were min, 60.37; max, 6158.77; and median, 2082.53. The results for CD8^+^IFN-γ^+^ cells were min, 41.51; max, 32146.13; median, 562.41; *n* = 9.

A summary of the results for 9 donor-independent expansions is shown in Figure 2E. Our data confirmed that, with the expansion system used, the final product contains antigen-specific IFN-γ-producing T cells (CD3^+^IFN-γ^+^ 25.3 ± 16.2%) with no significant differences between compartments (CD3^+^CD4^+^IFN-γ^+^, 15.7 ± 18.1%; CD3^+^CD8^+^IFN-γ^+^, 29.3 ± 20.3%; *p* = 0.3536). Moreover, significant differences were found regarding specificity gain in all subsets after 14-day of culture expansion (CD3^+^ *p* < 0.0001, CD3^+^CD4^+^ *p* < 0.0001, and CD3^+^CD8^+^ *p* < 0.0001).

Moreover, we analyzed the frequency of HLA-A^*^0201/NLVPMVATV pentamer^+^ CD8^+^ T cells. According to the results, cells expanded with pp65-pulsed DC show a higher percentage of antigen specific CD8^+^ T lymphocytes than pre-expansion cells (Supplementary Figure 4).

### CMV-Specific IFN-γ^+^ T Cells Are Effector Memory T Cells

To thoroughly characterize the expanded product, CD3^+^, CD3^+^CD4^+^, and CD3^+^CD8^+^ T cell populations were identified and classified into four different subsets based on the expression of CCR7 and CD45RA markers, following the gating strategy shown in Supplemental Figure 1. Here, naïve T cells (T_N_): CD45RA^+^CCR7^+^, central memory T cells (T_CM_): CD45RA^−^CCR7^+^, effector memory T cells (T_EM_): CD45RA^−^CCR7^−^, and terminally differentiated effector memory T cells (T_EMRA_): CD45RA^+^CCR7^−^.

**Figure 3:**
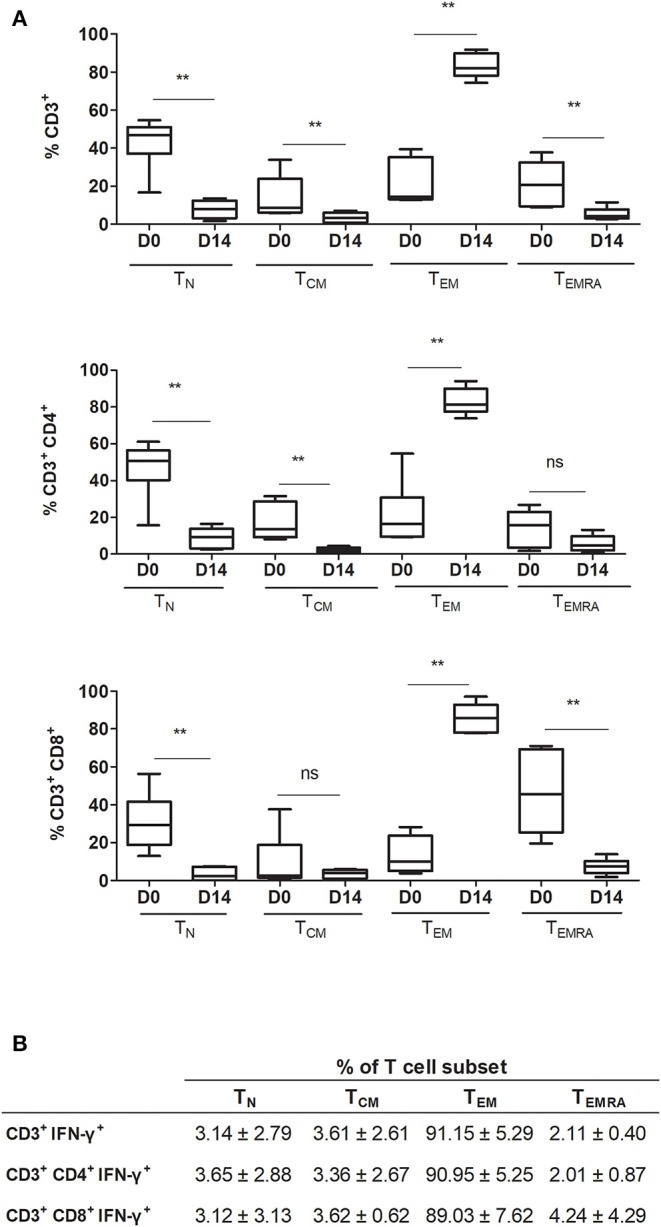
Frequency of different T cell differentiation subset phenotypes in the final product. **(A)** Comparison between pre-expansion and post-expansion for CD3^+^, CD3^+^CD4^+^, and CD3^+^CD8^+^ subpopulations (*n* = 6). **(B)** T cell differentiation phenotypes in post-expansion IFN-γ^+^ T cells (*n* = 4). T_N_ indicates naïve T cell (CCR7^+^CD45RA^+^); T_CM_, central memory T cell (CCR7^+^CD45RA^−^); T_EM_, effector memory T cell (CCR7^−^CD45RA^−^); and T_EMRA_, terminally differentiated effector memory T cell (CCR7^−^CD45RA^+^). **p < 0.01 (Mann-Whitney test).

After expansion, the T_N_ subset decreased significantly in all T cell populations (CD3^+^ *p* =0.0022, CD3^+^CD4^+^ *p* = 0.0043, and CD3^+^CD8^+^ *p* = 0.0022) (Figure 3A). T_EM_ increased significantly in CD3^+^, CD3^+^CD4^+^, and CD3^+^CD8^+^ cells (*p* = 0.0050, 0.0022, and 0.0022, respectively). In general, CD3^+^ subpopulations T_CM_ and T_EMRA_ decreased significantly after expansion (*p* = 0.0087). For CD3^+^CD4^+^ cells, the T_CM_ subset decreased significantly (*p* = 0.0022) and T_EMRA_ showed no significant changes (*p* = 0.1797). For CD3^+^CD8^+^ cells, the T_CM_ subset showed no significant changes (*p* = 0.9372) and the T_EMRA_ subset showed a significant decrease (*p* = 0.0022).

Interferon gamma, used here as a marker of CMV T cell specificity, was expressed by both CD3^+^CD4^+^ and CD3^+^CD8^+^ T cells. The T cell subsets expressing IFN-γ corresponded overwhelmingly to the T_EM_ subset: CD3^+^, 91.15 ± 5.29%; CD3^+^CD4^+^, 90.95 ± 5.25%; and CD3^+^CD8^+^, 89.03 ± 7.62% (Figure 3B). Moreover, when comparing the phenotype of pre-expanded cells, cells expanded with nonpulsed DC, and cells expanded with pp65-pulsed DC, we observed that the main T cell subset in specific expanded cells correspond to T_EM_ together with a higher expression of IFN-γ expression. Cells expanded with non-pulsed DC and pre-expansion cells, have a lower expression of IFN-γ and a higher percentage of T_N_ (Supplementary Figure 5). If we focus on the pp65-pulsed DC sample, and analyze the non-responsive cells (CD3^+^ IFN-γ^−^), the T cell subsets are distributed similarly to the responsive cells (CD3^+^ IFN-γ^+^) but with a lesser percentage of T_EM_, and a higher percentage of other T cell subsets, including T_N_ (Supplementary Figure 6).

Furthermore, the population of expanded cells that expressed PD1 corresponded to the IFN-γ^+^ population (Supplementary Figure 7).

### Functional and Specific Final Product Against pp65 CMV

**Figure 4:**
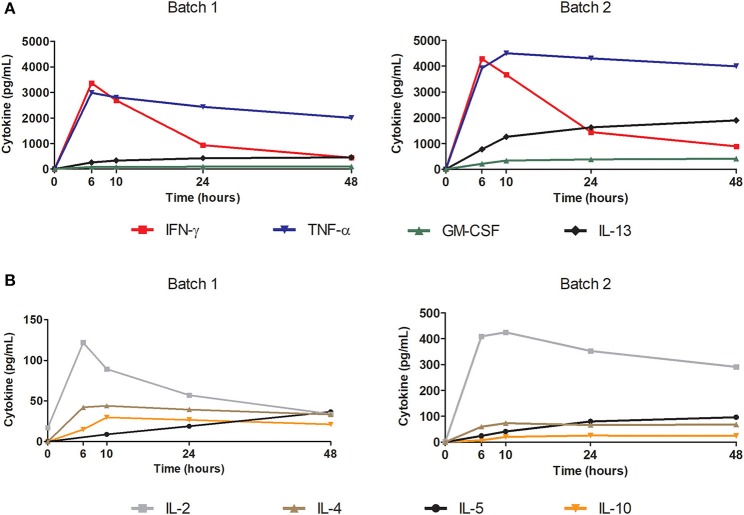
Polyfunctionality of the final thawed product tested using cytokine secretion kinetics after antigenic stimulation. Test performed with Luminex technology. Cytokines tested: IL-2, IL-4, IL-5, IL-10, IL-12 (p70), IL-13, IFN-γ, GM-CSF, and TNF-α. Cells were stimulated with pp65 50 ng/mL at a cell concentration of 1E+06 cells/mL in a 96-well plate. Graphs show the kinetics of the secreted cytokines in the supernatant (pg/mL) at each time: 0, 6, 10, 24, and 48 h. **(A)** Representation of secretion kinetics for cytokines IL-13, IFN-γ, GM-CSF, and TNF-α from 2 batches of final product. **(B)** Representation of secretion kinetics for cytokines IL-2, IL-4, IL-5, and IL-10 from 2 batches of final product.

Secretion kinetics of Th1 and Th2 cytokines after stimulation with pp65 of 2 cryopreserved batches of expanded cells at different time points (0, 6, 10, 24, and 48 h) are shown in Figure 4. Cytokine secretion had high concentrations of IFN-γ and TNF-α detected with a secretion peak after 6 h of stimulation. Furthermore, IL-2 was secreted in a similar manner but in much lower concentrations.

**Figure 5:**
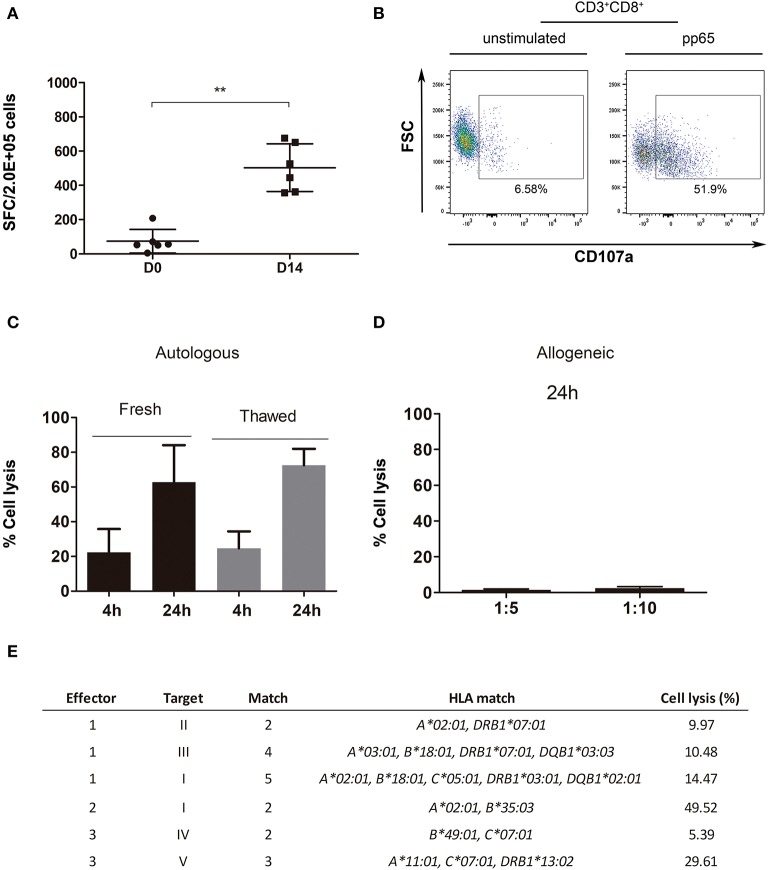
Potency and functional assays performed with the expanded product. **(A)** IFN-γ ELISPOT for D0 and D14 thawed cells (*n* = 6). **(B)** Example of a degranulation assay (CD8^+^CD107a^+^) dot plot for effector cells (VST), unstimulated (left plot) and stimulated with pp65 (right plot). **(C)** Cytotoxicity assay representation for pp65-pulsed autologous blasts specific lysis [4-h ratio, 1:5 (T:E); *n* = 3; 24-h ratio, 1:5 (T:E); *n* = 2]. **(D)** Cytotoxicity assay representation for irradiated allogeneic blasts unspecific cell lysis with 2–3 match (*n* = 6, fresh *n* = 2, thawed *n* = 4). **(E)** Cytotoxicity assay representation for effector cells (E): VST, cocultured with target cells (T), pp65-pulsed allogeneic blasts. Percentage of cell lysis calculated from a ratio of 1:10 (T:E). **p < 0.01 (Mann-Whitney test).

T cell response to pp65 was also assessed as a potency assay using IFN-γ ELISPOT for cryopreserved samples from day 0 and 14-day expanded product. Based on the number of spot forming colonies (SFC), a significant increase (*p* = 0.0022) in reactivity was detected after expansion (Figure 5A). A similar difference in reactivity was observed compared to the control of expanded cells with nonpulsed DC (Supplementary Figure 8).

A CD107a degranulation assay was performed to check the functionality of three final products. The expression of CD8^+^CD107a^+^ was detected in the presence of CMV pp65 peptide pool (Figure 5B), while only a slight expression was shown when cells were left unstimulated. However, this assay showed high variability between effector cell batches (CD8^+^CD107a^+^ 22.4 ± 25.71, *n* = 3, and CD4^+^CD107a^+^ 8.67 ± 11.74, *n* = 3) (Supplementary Figure 2).

We took advantage of the cytotoxicity assay to show that our product was able to induce cell death specifically in the target cell population (see gating strategy in Supplemental Figure 3). Both fresh and cryopreserved effector cells induced lysis among peptide-pulsed blasts, in a similar manner (fresh vs. cryopreserved: 4-h *p* = 0.7, 24-h *p* = 0.8). This further supports the feasibility of a ready-to-use cryopreserved cell-bank with verified post-thawing functional VSTs (Figure 5C). The expanded product in the presence of autologous blasts pulsed with the peptide resulted in a high percentage of cytotoxicity at both 4 and 24 h (23.45 ± 18.35% and 68.61 ± 19.76%, respectively).

We leveraged the cytotoxicity assay to assess *in vitro* safety by testing the alloreactivity and specificity of the final product. Supplementary Tables 1, 2 show, respectively, the high resolution HLA typing for expanded cells [used here as effector [E] cells] and PHA blasts [used here as target [T] cells]. Fresh and cryopreserved expanded cells were cocultured for 24 h with allogeneic blasts (with 2–3 HLA matches) at ratios of (T:E) 1:5 and 1:10 to test for alloreactivity (Figure 5D), which resulted in cell lysis below 5% in all cases. However, a correlation between the number of HLA mismatches and percentage of cell lysis was not observed (Pearson correlation: ratio 1:5, *p* = 0.8211; ratio 1:10, *p* = 0.9248; data not shown). Further characterization was performed by coculture of the final product with allogeneic blasts (2–5 HLA match), either pulsed with pp65 peptide pool or non-pulsed. Expanded T cells specifically induced cell lysis of the allogeneic blasts pulsed with pp65 regardless of the number of HLA matches (Figure 5E). Similarly, a correlation between the number of HLA matches and the percentage of cell lysis was not observed (Pearson test, *p* = 0.6460).

## Discussion

In recent years, expansion protocols for the creation of VST third-party banks for adoptive immunotherapy have been under development in different centers as an effective and feasible therapy for immunocompromised patients with herpesvirus infections (1, 5, 28–31). We showed a methodology based on *ex vivo* culture expansion of low-frequency VST to clinical numbers. The process is based on technology that allows scaling up and transfer to cGMP standards. Peripheral blood was used as starting material instead of leukapheresis, in order to facilitate donation compared to the direct selection protocol (32). Physiological antigen presenting cells, DC, are used as potent stimulators for specific T cell activation, together with an unspecific stimulation on day 5 based on anti-CD3 and anti-CD28 antibodies. Pp65 peptide pool was used to pulse DC for antigen specific stimulation due to being the most immunogenic protein of CMV (33). Nevertheless, immediate early 1 protein (IE-1) specific T cells have been demonstrated to have a protective effect (34). Therefore, and since the use of both pp65 and IE-1 peptides has been proven successful by Tzannou and colleagues (35), our future approach would be to use both peptides for the generation of CMV specific T cells. The protocol described in this study allowed us to produce *in vitro* safe and effective VST after a short expansion of only 14-day showing an advantage in time over protocols based on longer expansions (6, 33). The use of G-Rex technology enabled us to grow cells with a larger volume of media compared to the traditional plastic-based culture and therefore obtain a higher density. The final product was thoroughly characterized for IFN-γ expression among CD8^+^ cells, which are the cells that most express this cytokine, although the CD4^+^ subset is also essential for orchestrating the immune response (17, 18). According to the average of 42.2 fold-expansion obtained from the 9 samples, a single blood donation may yield up to 200 doses of 2E+07 VST/m^2^, an average fold expansion higher than other expansion protocols; which involves substantial cost reduction compared to other methodologies like direct selection. Enhancement of final product reactivity against CMV pp65 peptides was also confirmed by means of IFN-γ ELISPOT assays. The presence of T helper 1 (Th1) CD4^+^ cells (CD4^+^IFN-γ^+^), present in our final product, is associated with control of persistent infections (36). The proliferation of NK and B cells was prevented and, despite the use of IL-2, regulatory T cells were not present in the final product, thus facilitating the activity of the VSTs generated (37). Since naïve T cells are potentially alloreactive, it is also interesting that the final product contained low levels of T_N_ cells, thereby reducing the probability of GvHD (38). The final product is mainly composed of T_EM_, which provide the effector function needed. T_EM_ are known to mediate protective memory and they migrate to inflamed peripheral tissues and display immediate effector functions (39). The highest percentage of IFN-γ secretion was attributed to this subset, which can therefore rapidly control the infection. Despite the low concentration of T_CM_ in the final product, we hypothesize that these cells may engraft and confer long-lasting protection with later differentiation to effector cells upon antigenic stimulation. *In vivo* T cell persistence of third-party partially HLA-matched VST has been confirmed by other authors for up to 12 weeks (40). *Ex vivo* expanded cells could also be manufactured, in the case of hematopoietic stem cell transplantation, if available, from the same donor of the transplant, for which rates of functional VST engraftment have been shown to persist for up to 9 years (41). The option of *ex vivo* expansion on purpose for a single patient or in an autologous context is also conceivable. However, although personalized expansions probably have some advantages, such as choosing the highest HLA matching, this would require manufacturing time that is otherwise absent in a third-party bank.

Final product polyfunctionality was demonstrated by the detection of several secreted cytokines after cell stimulation. Among the inflammatory cytokines tested, we found not only IFN-γ after stimulation with pp65 but also high concentrations of TNF-α. Products with production of multiple cytokines have been shown to provide a more effective immune response against a pathogen (42).

We showed that the product has the potential to specifically kill pp65 peptide pool-pulsed autologous cells. To emulate allogeneic therapy conditions, we tested pp65-pulsed target cells with 2–3 match with our effector cells. The response varied from the HLA allele matched rather than with the number of HLA matching, in line with findings in the literature that certain pp65 epitopes are HLA restricted and some of them are immunodominant epitopes (33, 43–45). Moreover, the results shown by degranulation assays further confirmed that the VST obtained act as antigen-specific effector cells; cells degranulate, and therefore have the capacity to strike pp65 pulsed cells.

As for any other therapy it is essential that our product shows not only efficacy but safety. Alloreactivity assays were performed to characterize the *in vitro* safety of the product. When testing alloreactivity, we observed that effector cells did not lyse target cells that were not pulsed with the peptide pool, independently of the number of HLA matches. The similar functionality observed for both the fresh and cryopreserved product demonstrates the feasibility of generating a VST bank that maintains the product properties. The lack of correlation observed between HLA matching and specific cell lysis suggests that HLA restriction of immunodominant epitopes is more important than higher HLA compatibility between the donor and the patient in order to induce specific lysis. On the contrary, higher HLA matching would ensure engraftment of donor T lymphocytes as well as long-term protection. Alloreactivity is an important parameter to consider when using cell therapies as a treatment, as GvHD is one of the major concerns when infusing allogeneic cells. Approaches to avoid GvHD include donor-patient HLA matching and reducing the number of T_N_ cells present in the final product. Moreover, alloreactivity *in vitro* is reduced by *ex vivo* culturing with virus-specific stimuli, as unspecific cell lysis induced by the expanded cells is <5%. Our results complement the cytotoxicity assays performed, which ensured the ability of VST to kill target cells without attacking those cells not loaded with the antigen. Some reports indicate that virus-specific memory T cells can exert allo-HLA reactivity (46). Nevertheless, published studies report no increased incidence of GvHD, even when the donor had HLA mismatch, or toxicity related to VST infusion (1, 5, 8, 15, 29, 47).

Future perspectives include the translation of VST production and validation to cGMP standards. Future prospects also include the generation of other virus-specific T cells, such as EBV, AdV, and BKV, or even multivirus-specific T cells as shown feasible in the literature (5).

In summary, the use of pp65 peptide pool pulsed DCs and the addition of anti-CD3 and anti-CD28 antibodies in the presence of IL-2, IL-7, and IL-15 resulted in >98.89-fold CD3^+^ IFN-γ^+^ cell expansion at day 14. An average of 25.3% IFN-γ was achieved in 9 expansions, with very small amounts of CD56^+^, CD19^+^/CD20^+^, or CD4^+^CD25^+^FOXP3^+^ cells. T_EM_ cell subpopulations were increased after expansion, while T_N_ subpopulations were decreased in the final product. Moreover, the reactivity of the product against CMV peptide pp65 was enhanced after short culture expansion tested using the IFN-γ ELISPOT assay. In terms of effectivity and *in vitro* safety, strong cytotoxicity was shown when specific T cells were cocultured with pulsed pp65 autologous blasts. Final product degranulation was confirmed in line with cytotoxicity results. When expanded cells were cultured with non-pulsed allogeneic blasts, a maximum of 5% lysis was observed. Furthermore, cytotoxicity of expanded cells with pp65-pulsed allogeneic blasts showed specific cell lysis. In conclusion, we have shown a feasible and scalable process that can be easily transferred to cGMP standards and that generates a safe and functional product.

## Data Availability Statement

All datasets generated for this study are included in the article/Supplementary Material.

## Author Contributions

Experiments were designed by MG-V, ML-M, IO-V, and FR. Experimental data were generated by MG-V, ML-M, and IO-V, and analysis was performed by MG-V, ML-M, and EC. MG-V, ML-M, SQ, PB, and FR prepared the manuscript. All authors have reviewed the manuscript and have made substantial and intellectual contributions to the work.

### Conflict of Interest

PB declares having received honoraria from Amgen, Celgene, Gilead, Incyte, Jazz Pharmaceuticals, MSD, Novartis, Pfizer and Roche, not related with the present article. PB has received funding from the Carlos III FIS16/01433 Health Institute, Asociación Española contra el Cáncer (Ideas Semilla 2019) and a PERIS 2018-2020 grant from the Generalitat de Catalunya (BDNS357800). The remaining authors declare that the research was conducted in the absence of any commercial or financial relationships that could be construed as a potential conflict of interest.
