# Supplementary material for: Characterization of a Cytomegalovirus-Specific T Lymphocyte Product Obtained Through a Rapid and Scalable Production Process for Use in Adoptive Immunotherapy
Source: Front Immunol. 2020 Feb 25;11:271. doi: 10.3389/fimmu.2020.00271 (PMC7052482; doi:10.3389/fimmu.2020.00271)
Supplement: Supplementary file 1 [file Table_1.DOCX]

Supplementary Material

**Supplementary Table 1.** HLA typing at high resolution for effector samples used in the alloreactivity assay.

| **Batches** | **HLA type** |  |  |
| --- | --- | --- | --- |
| 1 | *A*02:01, A*03:01, B*18:01, B*51:08, C*05:01, C*16:02, DRB1*03:01, DRB1*07:01, DQB1*02:01, DQB1*03:03* |  |  |
| 2 | *A*02:01, A*03:01, B*35:03, B*51:01, C*04:01, C*16:01, DRB1*07:01, DRB1*12:01, DQB1*02:02, DQB1*03:01* | |  |
| 3 | *A*11:01, A*32:01, B*49:01, B*51:01, C*07:01, C*14:02, DRB1*13:02, DRB1*14:04, DQB1*05:03, DQB1*06:04* | |  |

**Supplementary Table 2.** HLA typing at high resolution for allogeneic PHA lymphoblasts used for alloreactivity assays.

| **Blasts** | **HLA type** |
| --- | --- |
| I | *A*02:01, A*23:01, B*18:01, B*35:03, C*05:01, C*12:03, DRB1*03:01, DRB1*04:08, DQB1*02:01, DQB1*03:04, DPB1*04:01, DPB1*04:01* |
| II | *A*01:01, A*02:01, B*44:03, B*56:01, C*01:02, C*04:01, DRB1*07:01, DRB1*11:01, DQB1*02:02, DQB1*03:01, DPB1*02:01, DPB1*04:01* |
| III | *A*03:01 A*24:02, B*18:01 B*35:08, C*04:01 C*07:01, DRB1*07:01 DRB1*13:02, DQB1*03:03 DQB1*06:04* |
| IV | *A*23:01, A*26:01, B*39:01, B*49:01, C*07:01, C*12:03, DRB1*01:01, DRB1*07:01, DQB1*02:02, DQB1*05:04* |
| V | *A*11:01, A*80:01, B*18:01, B*44:03, C*04:01, C*07:01, DRB1*13:02, DRB1*15:01, DRB3*03:01, DRB5*01:01 DQB1*06:02, DQB1*06:09, DPB1*04:01, DPB1*13:01* |


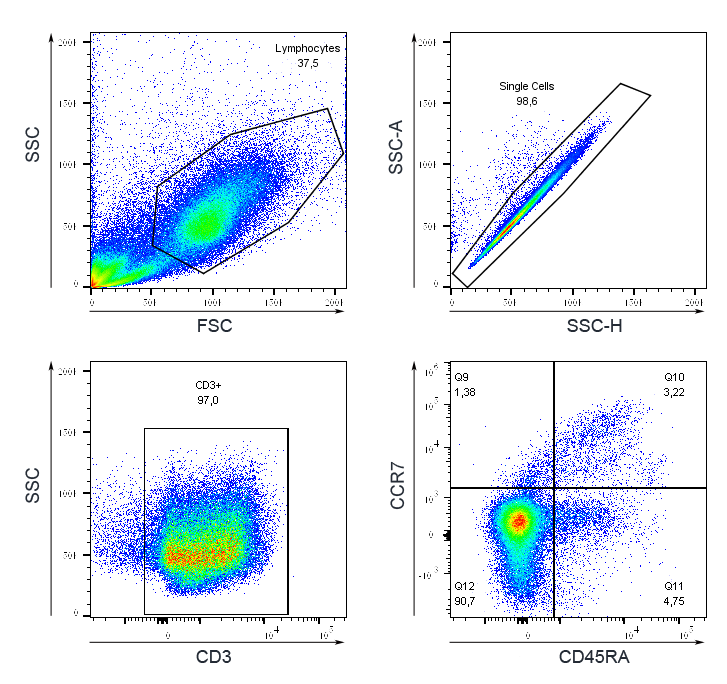


**Supplementary Figure 1.** Flow cytometry gating strategy to identify naïve (T_N_), central memory (T_CM_), effector memory (T_EM_), and terminally differentiated effector memory (T_EMRA_) T cell subsets. A dot plot for the lymphocyte selection by FSC and SSC was performed, followed by doublet discrimination. Classification of the population was done after CD3^+^ selection (from the singlets selection) by CD45RA and CCR7 gating, and determination of each cell population (T_N_: CD45RA^+^CCR7^+^, T_CM_: CD45RA^-^CCR7^+^, T_EM_: CD45RA^-^CCR7^-^, and T_EMRA_: CD45RA^+^CCR7^-^).


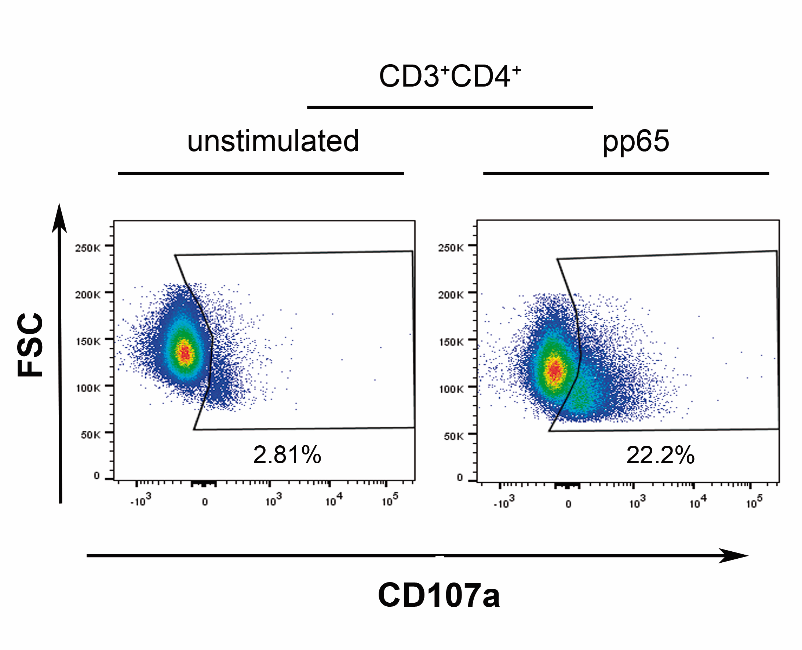


**Supplementary Figure 2.** Functional assays performed with the expanded product. Example of a degranulation assay (CD4^+^CD107a^+^) dot plot for effector cells (VST), unstimulated (left plot) and stimulated with pp65 (right plot).


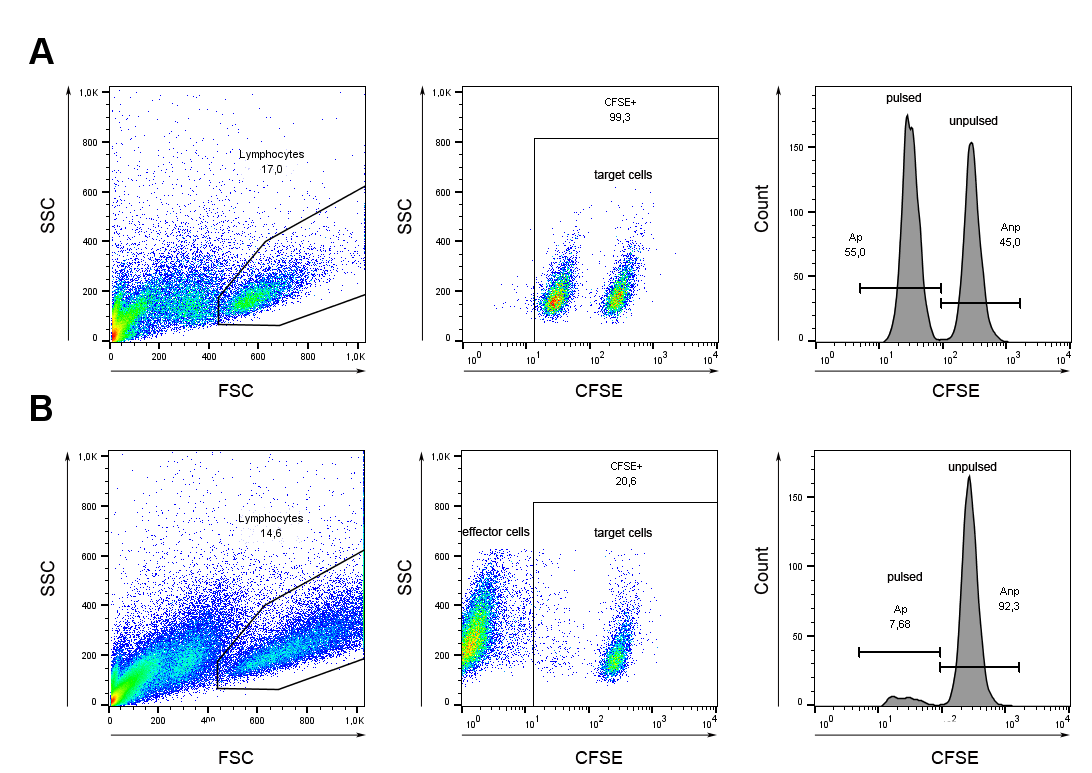


**Supplementary Figure 3.** Gating strategy for cytotoxicity assay. Initial lymphocyte population selection by FSC-H vs SSC-H. CFSE stained lymphocytes correspond to target cells: autologous pp65-pulsed (low CFSE concentration) and autologous not pulsed target cells (high CFSE concentration). (A) Control sample containing only target cells, pulsed and nonpulsed. (B) Effector sample with pulsed and nonpulsed cells, ratio 1:5 (T:E).

**
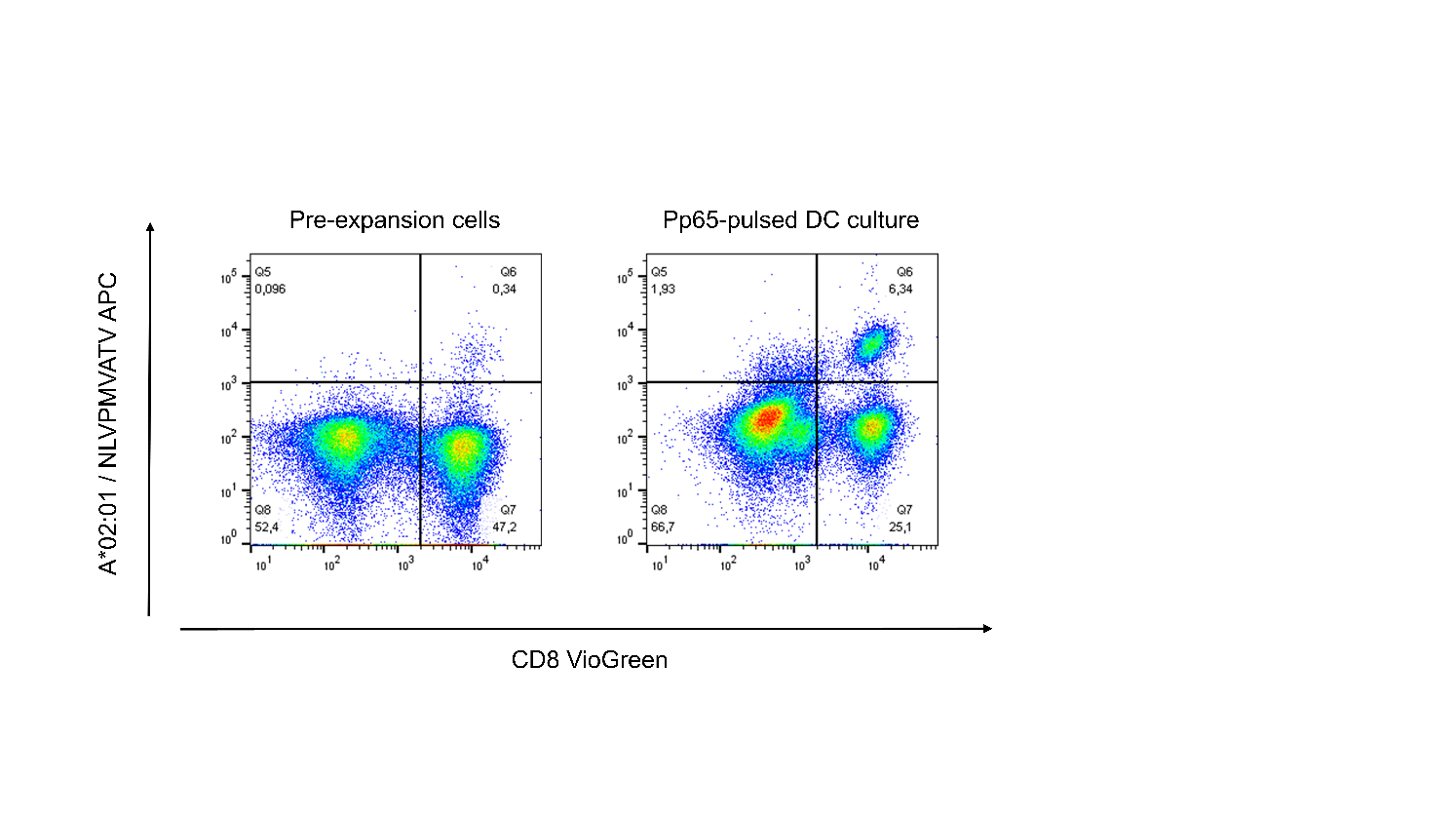
**

**Supplementary Figure 4.** Cell sample stained with HLA-A*02:01 Pentamer and antibody anti-CD8, samples from pre-expansion cells and cells expanded with pp65-pulsed DC.

**
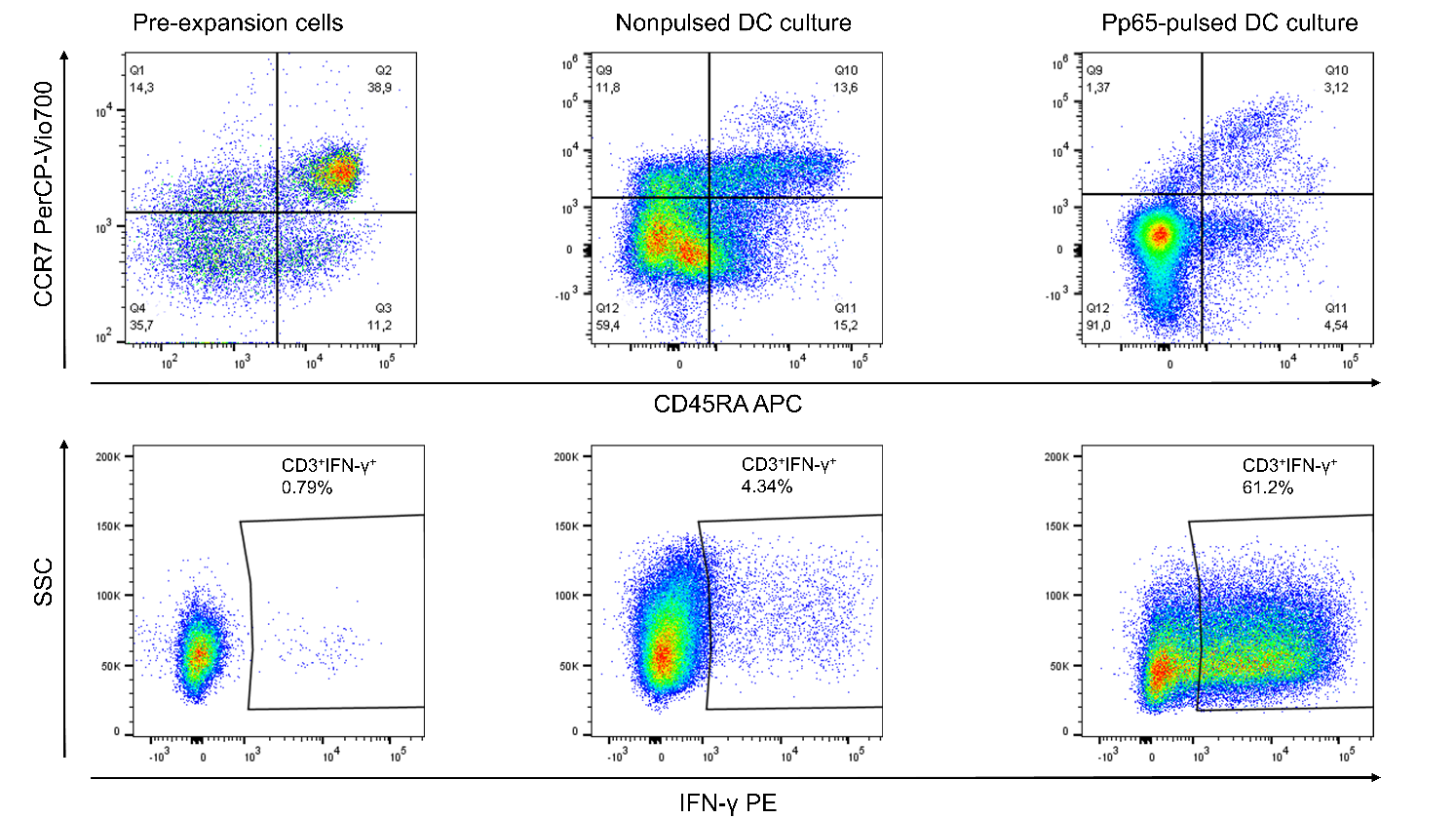
**

**Supplementary Figure 5.** T cell differentiation phenotypes and IFN-γ expression in pre-expansion and post-expansion T cells, both expanded with nonpulsed DC and pp65-pulsed DC. Naïve T cell (T_N_) correspond to CCR7^+^CD45RA^+^ population; central memory T cell (T_CM_) correspond to CCR7^+^CD45RA^-^ population; effector memory T cell (T_EM_) correspond to CCR7^-^CD45RA^-^ population; and terminally differentiated effector memory T cell (T_EMRA_) correspond to CCR7^-^CD45RA^+^ population. Gating strategy for IFN-γ secreting cells from one representative donor. Dot plots show the response of T lymphocytes against pp65 antigen stimulation.

**
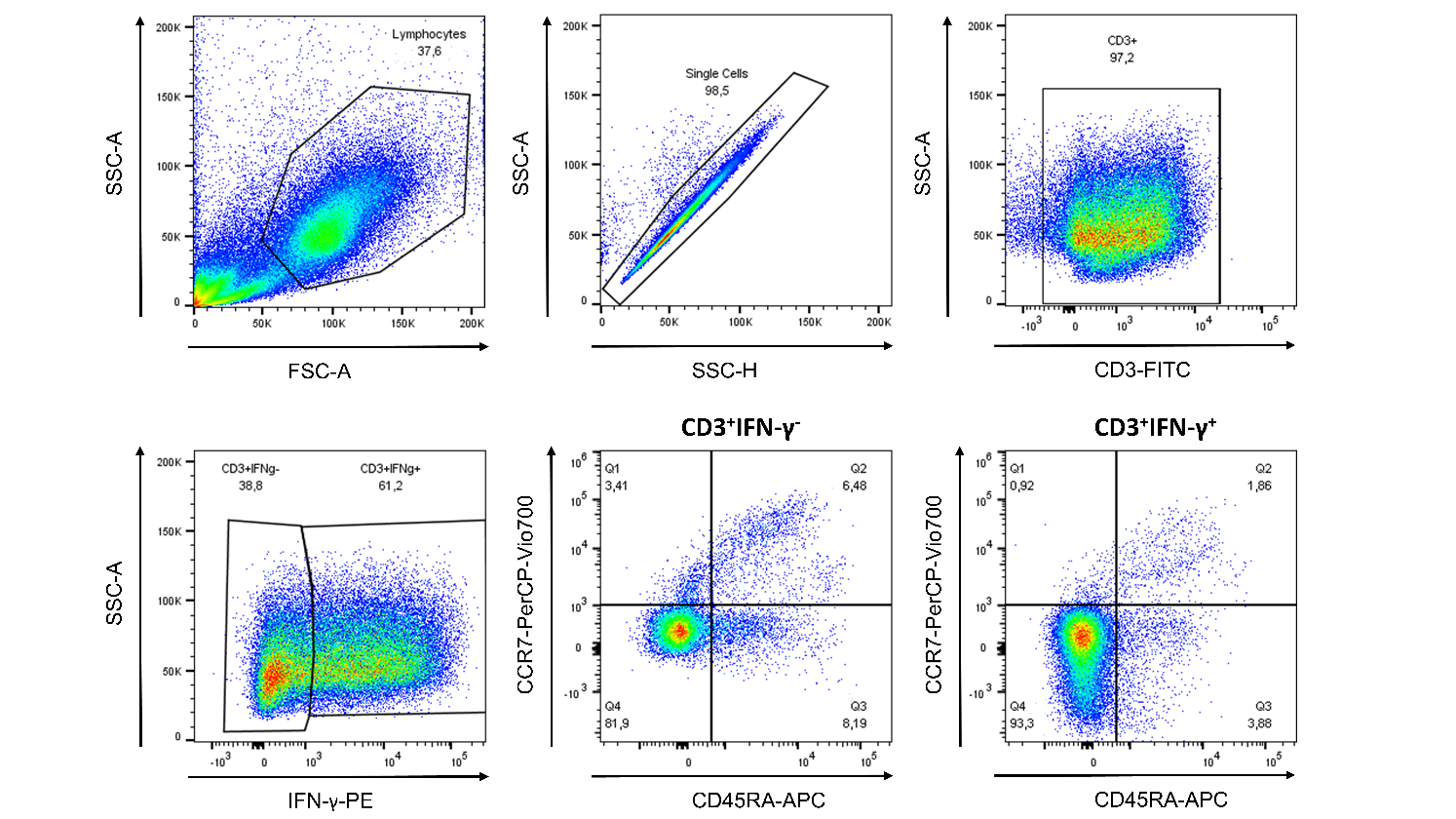
**

**Supplementary Figure 6.** Flow cytometry gating strategy to identify naïve (T_N_) (CCR7^+^CD45RA^+^), central memory (T_CM_) (CCR7^+^CD45RA^-^), effector memory (T_EM_) (CCR7^-^CD45RA^-^), and terminally differentiated effector memory (T_EMRA_) (CCR7^-^CD45RA^+^) T cell subsets from specific (CD3^+^IFN-γ^+^) and unspecific (CD3^+^IFN-γ^-^) T cell populations. Post-expansion sample cocultured with pp65 pool pulsed-DC for 14 days.

**
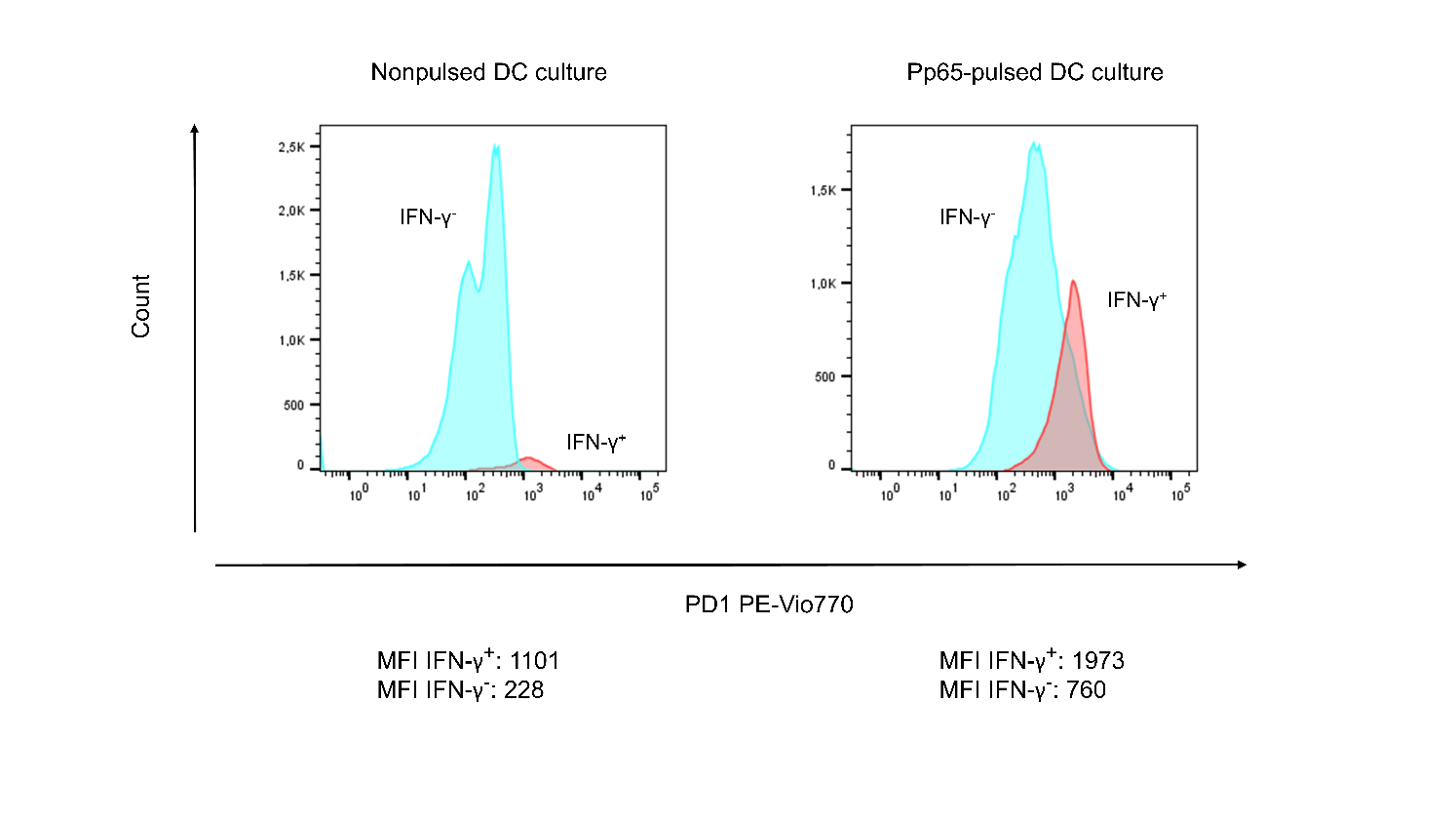
**

**Supplementary Figure 7.** Post-expansion T cells (D14) cultured with nonpulsed DC and pp65-pulsed DC culture. Histograms representation of PD1 expression from IFN-γ negative and positive T cells.

**
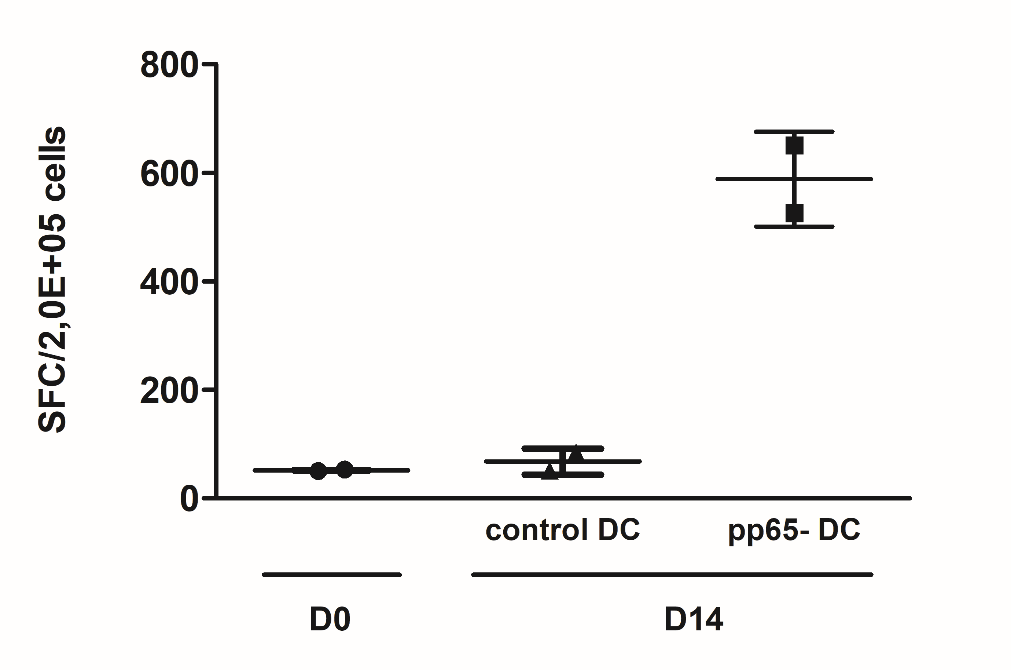
**

**Supplementary Figure 8.** IFN-γ ELISPOT for D0 and D14 thawed cells (n=2). Spot forming colonies from samples stimulated with peptide pool pp65. Samples from D14 correspond to cells expanded with nonpulsed DC (control DC) and pp65-pulsed DC (pp65-DC).
